# Impact of flow rates in a cardiac cycle on correlations between advanced human carotid plaque progression and mechanical flow shear stress and plaque wall stress

**DOI:** 10.1186/1475-925X-10-61

**Published:** 2011-07-19

**Authors:** Chun Yang, Gador Canton, Chun Yuan, Marina Ferguson, Thomas S Hatsukami, Dalin Tang

**Affiliations:** 1 School of Mathematical Sciences, Beijing Normal University, Lab of Math and Complex Systems, Ministry of Education, Beijing, China; 2 Mathematical Sciences Department, Worcester Polytechnic Institute, Worcester, MA 01609, USA; 3 Deparment of Radiology, University of Washington, Seattle, WA 98195, USA; 4 Division of Vascular Surgery, University of Washington, Seattle, WA. 98195, USA

## Abstract

**Background:**

Mechanical stresses are known to play important roles in atherosclerotic plaque initiation, progression and rupture. It has been well-accepted that atherosclerosis initiation and early progression correlate negatively with flow wall shear stresses (FSS). However, mechanisms governing *advanced *plaque progression are not well understood.

**Method:**

In vivo serial MRI data (patient follow-up) were acquired from 14 patients after informed consent. Each patient had 2-4 scans (scan interval: 18 months). Thirty-two scan pairs (baseline and follow-up scans) were formed with slices matched for model construction and analysis. Each scan pair had 4-10 matched slices which gave 400-1000 data points for analysis (100 points per slice on lumen). Point-wise plaque progression was defined as the wall thickness increase (WTI) at each data point. 3D computational models with fluid-structure interactions were constructed based on in vivo serial MRI data to extract flow shear stress and plaque wall stress (PWS) on all data points to quantify correlations between plaque progression and mechanical stresses (FSS and PWS). FSS and PWS data corresponding to both maximum and minimum flow rates in a cardiac cycle were used to investigate the impact of flow rates on those correlations.

**Results:**

Using follow-up scans and maximum flow rates, 19 out of 32 scan pairs showed a significant *positive *correlation between WTI and FSS (positive/negative/no significance correlation ratio = 19/9/4), and 26 out of 32 scan pairs showed a significant *negative *correlation between WTI and PWS (correlation ratio = 2/26/4). Corresponding to minimum flow rates, the correlation ratio for WTI vs. FSS and WTI vs. PWS were (20/7/5) and (2/26/4), respectively. Using baseline scans, the correlation ratios for WTI vs. FSS were (10/12/10) and (9/13/10) for maximum and minimum flow rates, respectively. The correlation ratios for WTI vs. PWS were the same (18/5/9), corresponding to maximum and minimum flow rates.

**Conclusion:**

Flow shear stress corresponding to the minimum flow rates in a cardiac cycle had slightly better correlation with WTI, compared to FSS corresponding to maximum flow rates. Choice of maximum or minimum flow rates had no impact on PWS correlations. Advanced plaque progression correlated positively with flow shear stress and negatively with plaque wall stress using follow-up scans. Correlation results using FSS at the baseline scan were inconclusive.

## Introduction

Cardiovascular diseases are the Number One cause of death in the developed countries and are becoming the Number One cause of death worldwide. Most cardiovascular diseases are related to atherosclerotic plaques whose rupture often leads to severe clinical event such as heart attack and stroke. It is of ultimate importance that we could understand the mechanisms governing plaque progression and rupture processes, and predict the drastic events (rupture, heart attack, and stroke) before their actual happening. It has been well accepted that low and oscillating blood flow shear stresses (LFSS) correlate positively with intimal thickening and atherosclerosis initiation [1-8]. However, results for advanced plaque progression based on patient-tracking data are relatively rare in the literature. Tang et al. used 2D structure-only models based on in vivo MRI patient-tracking data from 21 patients and their results indicated that 18 out of 21 patients studied showed significant negative correlation between plaque progression measured by wall thickness increase (WTI) and plaque wall stress (PWS, structure maximum principal stress taken at lumen wall) at follow-up time (T2). The 95% confidence interval for the Pearson correlation coefficient was (-0.443, -0.246), p < 0.0001 [9]. A recent paper from the same group reported that advanced human carotid plaque progression correlates positively with flow shear stress (FSS) using follow-up MRI scan data [10]. 3D models with fluid-structure interactions based on in vivo magnetic resonance images (MRI) of carotid plaques from 14 consented patients and 32 MRI scan pairs (baseline and follow-up) were used in that study. Two important observations from the study could be made: a) correlations between plaque progression with mechanical stresses (PWS and FSS) for advanced plaques may be different from those for plaques at their initiation and early development stage; b) correlations using follow-up time point data may be different from those using baseline time point data. Since it is generally believed that lower flow shear stress may promote plaque progression and our earlier analyses were performed only for data corresponding to the peak flow rate in a cardiac cycle. In an attempt to quantify the impact of flow rate on plaque progression, flow shear stress on the lumen corresponding to maximum and minimum flow rates in a cardiac cycle were calculated and their correlations with plaque progression were compared to investigate the flow rate impact. Correlation results for plaque wall stress were also reported.

## Methods

The patient MRI data, 3D FSI model construction, solution methods, node type and data point selection procedures were the same as those reported in [10] and it is briefly outlined here.

### *In vivo *serial MRI data acquisition and segmentation

**Figure 1:**
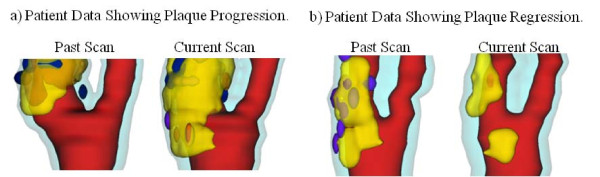
**3D plaque samples re-constructed from in vivo MR images showing progression and regression**. (a): one sample showing plaque growth; (b): one sample showing plaque reduction. Time interval: 18 months. Red: lumen; Yellow: lipid; Dark blue: calcification; light blue: outer wall.

After informed consent, serial MRI data of carotid atherosclerotic plaques from 14 patients (13 male, 1 female; age: 59-81, mean = 71.9) were acquired 2-4 times (scan interval: 18 months) by the University of Washington Vascular Imaging Laboratory using approved protocols. All patients had advanced atherosclerosis with 75% averaged stenosis severity by area (50% by diameter). Multi-contrast MRI scans were conducted on a GE SIGNA 1.5-T whole body scanner using an established protocol [10]. A custom-designed computer package CASCADE (Computer-Aided System for Cardiovascular Disease Evaluation) developed by the Vascular Imaging Laboratory (VIL) at the University of Washington (UW) was used to perform image analysis and segmentation. The slice thickness was 2 mm. Field of view = 160 mm × 160 mm. Matrix size 512 × 512 (the real matrix size was 256 × 256. Images were machine interpolated to 512 × 512). After interpolation, the in-plane resolution was 0.31 × 0.31 mm^2^. Thirty-two scan-pairs (called baseline and follow-up scans) were formed for this study, with MRI slices matched for each pair. Figure 1 gives two examples re-constructed from MRI data showing plaque progression and regression.

A patient with 4 scans gave 3 scan pairs (Scan 1, Scan 2), (Scan 2, Scan 3), and (Scan 3, Scan 4). Scan pairs for patients with 2 or 3 scans were formed similarly. Once the pairs were formed, they were all treated equal. Scans in each pair were referred to as (baseline scan, follow-up scan), or (Time 1 scan, Time 2 scan). Time 1 (T1) and Time 2 (T2) were used for easy reference.

### 3D Fluid-structure interaction plaque model construction and solution methods

**Figure 2:**
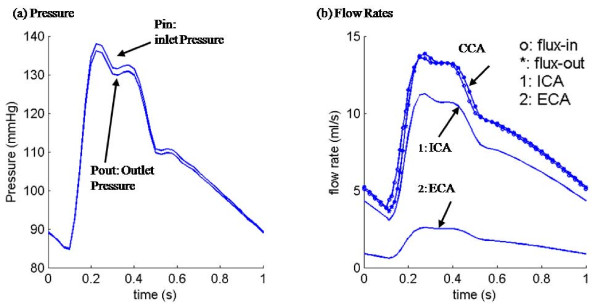
**Pressure conditions specified at the inlet (CCA) and outlet (ICA and ECA) in one case and the flow rates obtained from the FSI plaque model**.

3D plaque models with fluid-structure interactions (FSI) were constructed for all the plaques based on in vivo MRI data using established procedures [10], including plaque material properties and pressure conditions. Blood flow was assumed to be laminar, Newtonian, viscous and incompressible. A typical pressure profile and associated flow rates in one cardiac cycle for one plaque sample were provided in Figure 2[3]. Patient-specific systolic and diastolic pressure conditions from the last hospital admission were used as the maximum and minimum of the imposed pulsatile pressure waveforms at the inlet and outlet of the artery. The pressure profile in Figure 2 was adjusted for each patient using patient-specific systolic and diastolic arm pressure conditions. No patient-specific flow rate information was available. So the inlet and outlet pressure profiles (see Figure 2) were adjusted the same way for all patients, kept pressure drop proportional to arm pressure conditions. Flow rates for each patient were dependent on pressure at the inlet and outlet, stenosis severity, and vessel material properties. These modeling assumptions were about the best we could do with the limited information available to us. Since the focus of this study was to determine the correlations between mechanical forces (FSS and PWS) and plaque progression, the artery wall in the FSI model was assumed to be uniform, hyperelastic, isotropic, incompressible and homogeneous. The nonlinear modified Mooney-Rivlin model was used to describe the material properties of the vessel wall [10-13]. The strain energy function was given by,(1)(2)

where I_1 _and I_2 _are the first and second strain invariants, **C **= [C_ij_] = **X**^T^**X **is the right Cauchy-Green deformation tensor, **X **= [X_ij_] = [∂x_i_/∂a_j_], (x_i_) is the current position, (a_i_) is the original position, c_i _and D_i _are material parameters chosen to match experimental measurements and the current literature [10]. Parameter values used for the arterial vessel wall in this model were: c_1 _= 368000 dyn/cm^2^, c_2 _= 0, D_1 _= 144000 dyn/cm^2^, D_2 _= 2.0.

For FSI models based on in vivo MRI data, a shrink-stretch process was needed to obtain the no-load starting geometry and match in vivo geometry under pressurized and stretched condition. The shrinkage in axial direction was 9% so that the vessel would regain its *in vivo *length with a 10% axial stretch. Circumferential shrinkage for lumen (about 8-12%) and outer wall (about 2-5%) was determined by trial-and-error so that: 1) total mass of the vessel was conserved; 2) the loaded plaque geometry after 10% axial stretch and pressurization had the best match with the original *in vivo *geometry.

The 3D FSI models were solved by ADINA, using unstructured finite element methods for both fluid and solid domains. Nonlinear incremental iterative procedures were used to handle fluid-structure interactions. The governing finite element equations for both solid and fluid models were solved by Newton-Raphson iteration method. More details of the computational models and solution methods can be found in Tang et al. [13-15], Yang et al. [10] and Bathe [11]. Plaque wall stress and flow shear stress data corresponding to peak systolic pressure were recorded for analysis.

### Plaque progression measurements and data extraction for correlation analysis

**Figure 3:**
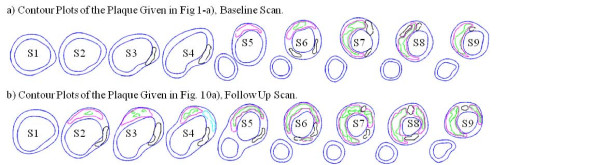
**Segmented contour plots of the plaque given in a)**. Black: Calcification; Magenta: lipid core. Some hemorrhage (green) was found inside lipid cores.

For each scan pair, slices from the baseline (Time 1, or T1) and follow-up (Time 2, or T2) scans were matched using the carotid bifurcation as the registration reference (Figure 3). Only matched common carotid artery (CCA) and internal carotid artery (ICA) slices were chosen for analysis since external carotid arteries (ECA) are less prone to atherosclerosis. For each matched slice, 100 evenly-spaced points from the lumen were selected and vessel wall thickness, PWS, and FSS from 3D FSI model solutions at each point for Time 1 and Time 2 were obtained for analysis. For the 32 pairs, 400-1000 matched data points for each plaque were obtained for correlation analysis. Plaque progression at each data point was expressed by vessel wall thickness increase (WTI) defined as(3)

In view of the fact that advanced plaques have irregular geometries, a piecewise equal-step method was introduced to calculate wall thickness at each data point [10]. This method is better than the shortest-distance method used in our previous paper [9].

To calculate the traditional wall flow shear stress (FSS) in the longitudinal direction, for a given lumen point ***x***, the tangential surface *Γ *and normal direction ***n ***to the lumen surface at that point were determined first. Let ***σ ***be the flow stress tensor, then the flow stress vector acting on *Γ *at point ***x ***is given by:(4)

Let ***t ***be the unit tangent vector on *Γ *at ***x ***in the longitudinal direction, then the flow shear in the longitudinal direction is given by(5)

WTI, PWS and FSS were extracted from all data points for all the 32 pairs corresponding to both maximum and minimum flow rates in a cardiac cycle. The phrase "in a cardiac cycle" will be omitted when referring to maximum or minimum flow rates when no confusion arises. Standard statistical linear regression analysis was performed to quantify the correlation between plaque progression measured by WTI and both M-FSS and FSS at baseline and follow-up scans.

## Results

Correlation results for wall flow shear stress (FSS) and plaque wall stress (PWS) with plaque progression (WTI) were obtained for Time 1 (baseline) and Time 2 (follow up) corresponding to maximum and minimum flow rates and are summarized in Table 1 and Table 2. Details are reported below.

**Table 1:** Summary of correlation results between flow shear stress (FSS) and plaque progression from 32 scan pairs using both baseline and follow-up scans and corresponding to maximum and minimum flow rates in a cardiac cycle.

Case #	# of Data Pts	FSS Baseline Max-Q		FSS Baseline Min-Q		FSS Follow-Up Max-Q		FSS Follow-Up Min-Q	
		
		r	p	r	p	r	p	r	p
C1	400	*0.015*	0.761	*0.025*	0.619	*0.125*	0.013	*0.130*	0.009

C2	400	*-0.108*	0.032	*-0.110*	0.027	*0.058*	0.247	*0.057*	0.256

C3	900	*0.338*	< .001	*0.337*	< .001	*0.401*	< .001	*0.403*	< .000

C4	400	*-0.450*	< .001	*-0.460*	< .001	*-0.144*	0.004	*-0.173*	0.001

C5	800	*0.170*	< .001	*0.181*	< .001	*0.432*	< .000	*0.439*	< .000

C6	800	*-0.379*	< .001	*-0.384*	< .001	*0.123*	0.001	*0.114*	0.001

C7	800	*0.117*	0.001	*0.108*	0.002	*0.125*	< .001	*0.126*	< .001

C8	800	*-0.088*	0.013	*-0.090*	0.011	*0.183*	< .001	*0.181*	< .001

C9	600	*-0.305*	< .001	*-0.300*	< .001	*-0.288*	< .001	*-0.293*	< .001

C10	600	*0.392*	< .001	*0.391*	< .001	*0.348*	< .001	*0.352*	< .001

C11	700	*-0.313*	< .001	*-0.307*	< .001	*-0.311*	< .001	*-0.312*	< .001

C12	700	*0.005*	0.887	*0.008*	0.834	***-0.077***	**0.043**	***-0.072***	**0.056**

C13	600	*-0.117*	0.004	*-0.110*	0.007	*-0.027*	0.513	*-0.013*	0.745

C14	1000	*0.188*	< .001	*0.193*	< .001	*0.337*	< .001	*0.340*	< .001

C15	1000	*0.108*	< .001	*0.102*	0.001	*0.257*	< .001	*0.252*	< .001

C16	900	*0.007*	0.845	*-0.010*	0.760	***-0.146***	**< .001**	***0.310***	**< .001**

C17	800	***0.188***	**< .001**	***-0.199***	**< .001**	*0.273*	< .001	*0.288*	< .001

C18	800	*0.056*	0.116	*0.054*	0.128	*0.154*	< .001	*0.159*	< .001

C19	800	*-0.032*	0.361	*-0.025*	0.484	*0.274*	< .001	*0.283*	< .001

C20	800	*-0.587*	< .001	*-0.588*	< .001	*-0.468*	< .001	*-0.463*	< .001

C21	900	*0.135*	< .001	*0.134*	< .001	*0.139*	< .001	*0.137*	< .001

C22	900	*-0.036*	0.282	*-0.053*	0.111	*0.006*	0.858	*0.002*	0.947

C23	700	*-0.051*	0.182	*-0.041*	0.281	*0.263*	< .001	*0.272*	< .001

C24	800	*0.028*	0.436	*0.021*	0.548	*0.082*	0.020	*0.081*	0.022

C25	800	*-0.021*	0.547	*-0.029*	0.421	*0.193*	< .001	*0.203*	< .001

C26	700	*0.054*	0.150	*0.058*	0.128	*0.153*	< .001	*0.162*	< .001

C27	700	*-0.382*	< .001	*-0.389*	< .001	*-0.044*	0.246	*-0.037*	0.329

C28	900	*-0.369*	< .001	*-0.373*	< .001	*-0.223*	< .001	*-0.225*	< .001

C29	900	*0.207*	< .001	*0.215*	< .001	*0.207*	< .001	*0.205*	< .001

C30	800	*0.332*	< .001	*0.336*	< .001	*0.361*	< .001	*0.364*	< .001

C31	800	*-0.420*	< .001	*-0.418*	< .001	*-0.357*	< .001	*-0.354*	< .001

C32	800	*-0.489*	< .001	*-0.498*	< .001	*-0.170*	< .001	*-0.175*	< .001

Positive		10		9		19		20	

Negative		12		13		9		7	

No Signifi.		10		10		4		5	

**95% CI**		**(-0.15, 0.037)**		**(-.163, 0.024)**		**(-0.015, 0.155)**		**(0.01, 0.172)**	

New correlation results from the 32 pairs indicated that advanced plaque progression (WTI) had positive correlation with FSS from follow-up scans. The correlation results using the baseline scan were inconclusive.

**Table 2:** Summary of correlation results between plaque wall stress (PWS) and plaque progression from 32 scan pairs using both baseline/follow-up scans and maximum and minimum flow rates in a cardiac cycle.

Case #	# of Data Pts	PWS Baseline Max-Q		PWS Baseline Min-Q		PWS Follow-Up Max-Q		PWS Follow-Up Min-Q	
		
		r	p	r	p	r	p	r	p
C1	400	-0.060	0.232	-0.059	0.243	-0.301	< .001	-0.299	< .001

C2	400	0.192	< .001	0.192	< .001	-0.209	< .001	-0.209	< .001

C3	900	0.019	0.564	0.022	0.505	-0.440	< .001	-0.437	< .001

C4	400	0.467	< .001	0.469	< .001	-0.079	0.116	-0.085	0.091

C5	800	0.270	< .001	0.260	< .001	-0.108	0.002	-0.129	< .001

C6	800	-0.039	0.274	-0.023	0.508	-0.279	< .001	-0.275	< .001

C7	800	0.133	< .001	0.145	< .001	-0.179	< .001	-0.166	< .001

C8	800	0.267	< .001	0.263	< .001	-0.133	< .001	-0.141	< .001

C9	600	0.457	< .001	0.457	< .001	0.208	< .001	0.208	< .001

C10	600	-0.297	< .001	-0.297	< .001	-0.622	< .001	-0.622	< .001

C11	700	-0.048	0.208	-0.043	0.252	-0.325	< .001	-0.329	< .001

C12	700	-0.024	0.530	-0.015	0.695	-0.228	< .001	-0.218	< .001

C13	600	-0.119	0.004	-0.120	0.003	-0.229	< .001	-0.242	< .001

C14	1000	-0.098	0.002	-0.097	0.002	-0.341	< .001	-0.342	< .001

C15	1000	0.235	< .001	0.240	< .001	-0.101	0.001	-0.100	0.002

C16	900	-0.037	0.269	-0.037	0.262	-0.302	< .001	-0.307	< .001

C17	800	-0.125	< .001	-0.121	< .001	-0.386	< .001	-0.383	< .001

C18	800	0.030	0.397	0.030	0.396	-0.368	< .001	-0.369	< .001

C19	800	-0.017	0.632	-0.016	0.649	-0.228	< .001	-0.228	< .001

C20	800	0.492	< .001	0.491	< .001	0.253	< .001	0.252	< .001

C21	900	0.097	0.004	0.094	0.005	-0.080	0.016	-0.081	0.015

C22	900	-0.135	< .001	-0.135	< .001	-0.241	< .001	-0.241	< .001

C23	700	0.408	< .001	0.412	< .001	-0.144	< .001	-0.143	< .001

C24	800	0.242	< .001	0.242	< .001	-0.402	< .001	-0.403	< .001

C25	800	0.227	< .001	0.226	< .001	-0.374	< .001	-0.375	< .001

C26	700	0.107	0.005	0.107	0.005	-0.221	< .001	-0.220	< .001

C27	700	0.024	0.531	0.019	0.609	-0.416	< .001	-0.415	< .001

C28	900	0.470	< .001	0.470	< .001	-0.011	0.741	-0.014	0.673

C29	900	0.080	0.017	0.087	0.009	0.013	0.694	0.016	0.639

C30	800	0.110	0.002	0.112	0.002	-0.052	0.140	-0.051	0.152

C31	800	0.127	< .001	0.125	< .001	-0.149	< .001	-0.154	< .001

C32	800	0.119	0.001	0.117	< .001	-0.158	< .001	-0.161	< .001

Positive		18		18		2		2	

Negative		5		5		26		26	

No Signifi.		9		9		4		4	

**95% CI**		**(0.039, 0.184)**		**(0.041, 0.185)**		**(-0.273, -0.142)**		**(-0.273, -0.143)**	

New correlation results from the 32 pairs indicated that advanced plaque progression correlated with PWS negatively using follow-up scans and positively using baseline scans. Flow rates had almost no impact on those correlations.

### Wall flow shear stress (FSS) correlates positively with wall thickness increase (WTI) using time 2 data, maximum and minimum flow

Table 1 summarizes correlation results between WTI and FSS at Time 1 and Time 2. Using FSS at Time 2 with maximum flow rate, statistically significant positive correlation between WTI and FSS was found in 19 of the 32 cases examined (9 negative, 4 no significance). The 95% confidence interval (CI) for Pearson correlation (PC) coefficient values was (-0.015, 0.155). The correlation ratio (positive: negativess: no significance) was (20:7:5) corresponding to the minimum flow rate. The 95% confidence interval (CI) was (0.01, 0.172). The correlation results corresponding to minimum flow rates were slightly better than those for maximum flow rates. Only 1 case (C16) changed from negative correlation under maximum flow to positive correlation under negative flow. And the p-value for one case changed from 0.043 to 0.056 (therefore became no significance). The correlations were weak in general and results were very close using either maximum or minimum flows.

### No significant correlation between FSS and WTI using time 1 data, maximum and minimum flow

The positive, negative and no significance correlation cases between WTI and FSS were 10, 12, and 10 out of 32 cases corresponding to maximum flow rates, respectively. The 95% confidence interval (CI) was (-0.15, 0.037). Using minimum flow rates, the correlation ratio was (9:13:10) with 95% CI interval (-0.163, 0.024). Correlation results for both maximum and minimum flow rates were very similar; with minimum flow rates gave slightly better results. Only 1 case (C17) changed from positive correlation under maximum flow to negative correlation under minimum flow.

### Plaque wall stress (PWS) correlates negatively with wall thickness increase (WTI) using time 2 data, maximum and minimum flow

Table 2 summarizes correlation results between PWS and WTI at Time 1 and Time 2. Using PWS at Time 2 with maximum flow rate, statistically significant negative correlation between PWS and FSS was found in 26 of the 32 cases examined (2 positive, 4 no significance). The 95% confidence interval (CI) for Pearson correlation (PC) coefficient values was (-0.273, -0.142). The correlation ratio and 95% CI interval corresponding to the minimum flow rate was exactly the same. That was not surprising since changing flow rates (actually it was the change of pressure for PWS) only caused proportional change in PWS which did not change correlation results.

### Plaque wall stress (PWS) correlates positively with WTI using time 1 data, maximum and minimum flow

The positive, negative and no significance correlation cases between PWS and WTI and FSS were 18, 5, and 9, respectively, corresponding to both maximum and minimum flow rates. The 95% confidence interval (CI) corresponding to maximum and minimum flow rates were (0.039, 0.184) and (0.041, 0.185), respectively.

## Discussion

An attempt was made to quantify impact of flow rates in a cardiac cycle on correlations between plaque progression and mechanical stresses. Correlation ratio for FSS vs. WTI corresponding to minimum flow rate at follow-up was (20:7:5), better than (19:9:4) corresponding to maximum flow rate. Overall, our results from the 32 pairs indicated that there is a positive correlation between advanced carotid plaque progression and flow shear stress using follow-up scan data, corresponding to both maximum and minimum flow rates. The correlation between plaque wall stress (PWS) and WTI was negative using follow-up data and positive using baseline data. Flow rate had almost no impact on correlations between PWS and WTI. The study using baseline FSS data for possible correlation between advanced carotid plaque progression and flow shear stress was inconclusive. All of these weak or inclusive correlation results suggest that more detailed data analysis [3,4] may be needed to discover localized plaque progression and mechanical stress (FSS and PWS) behaviors that the overall correlation analysis could not reveal. It also suggests that non-mechanical factors such as cellular activities, chemical factors (medication), genetic factors (genes), and diseases (diabetes, high cholesterol, high blood pressure) may contribute to plaque progression and their impact should be investigated.

Very few correlation studies for plaque progression using patient follow-up could be found in the current literature, mainly due to the fact that it is expensive to conduct large-scale patient studies and it takes a long time to observe plaque progression. Gibson et al. (1993) studied 20 human coronary arteries (time interval: 3 years) and found that there were negative correlations between flow shear stress at baseline and vessel diameter changes (15 negative correlation, 5 no significance) [4]. They did not use vessel wall thickness change as plaque progression measurements. Wentzel et al. (2005) used serial MRI (time interval: 24 months) to investigate the role of FSS in plaque progression and regression in the thoracic aorta. Ten patients participated in their study. Velocity was measured at each 2 cm starting from the arch using phase-contrast MRI and FSS was calculated based on pc-MRI measurements. Each cross-section was divided into 4 quadrants and wall thickness of each quadrants at baseline and follow-up were calculated. Each patient had 16 locations (4 segments × 4 quadrants) for analysis. Their results indicated that FSS at baseline was a good predictor for wall thickness (which was not a surprise since WT correlated strongly with stenosis severity), but did not predict plaque regression. This is consistent with our findings.

One difference between the current paper and our previous one [10] is that the traditional wall flow shear stress (FSS) in the longitudinal direction was used in this paper (See 2.3) to be more in line with the community, while flow maximum shear stress (FMSS) has been used on all our previous publications [10,13-15]. FMSS is defined as the maximum of shear stresses along all tangential directions on the lumen surface at the selected point and is more suitable for 3D models with complex flow behaviors. FMSS is provided by ADINA so it is also easier to use. The traditional wall flow shear stress (FSS) is used in this paper as our effort to further indicate that our previous correlation conclusions remained to be true when traditional wall FSS is used. Table 3 indicated that correlation results using FMSS and FSS were in good agreement. Overall, the correlations were weak. Differences caused by a) using different flow rates in a cardiac cycle; b) using traditional FSS calculations were very minor.

**Table 3:** Comparison of correlation results using flow maximum shear stress (FMSS) and traditional flow wall shear stress (FSS).

	FMSS Baseline Max-Q	FMSS Follow-Up Max-Q	FSS Baseline Max-Q	FSS Follow-Up Max-Q
Positive	**10**	**21**	10	19

Negative	**15**	**8**	12	9

No Signifi.	**7**	**3**	10	4

**95% CI**	**(-0.167, 0.025)**	**(0.012, 0.187)**	**(-0.15, 0.037)**	**(-0.015, 0.155)**

Modeling limitations include the following items: **a) **arm cuff systolic/diastolic pressures were used since on-site pressures were not available; **b) **isotropic material properties from the literature were used for the vessel since no patient-specific material properties were available. Layer-specific anisotropic material properties were not used in our models [16]; **c) **to reduce the model construction effort, plaque components were not included in the vessel wall. This certainly effected the accuracy of stress calculations and error estimates were given in [10]; d) blood flow was assumed laminar because the average stenosis severity (by diameter) of the 47 plaques was 50% and laminar flow assumption could be accepted [17].

## Conclusion

Lower flow rates in a cardiac cycle led to slightly better correlation between WTI and FSS, but had no impact on PWS correlation. Advanced plaque progression correlated positively with flow shear stress and negatively with plaque wall stress using follow-up scans. Correlation results using FSS at the baseline scan were inconclusive.

## Competing interests

Other than the grants listed in the acknowledgement section, the authors declare that they have no other competing interest.

## Authors' contributions

CYg and DT were responsible for computational modeling and data analysis part. CYn, GC, TH and MF were responsible for the MRI data and histology data acquisition and the segmentation part. All authors 1) have made substantial contributions to conception and design, or acquisition of data, or analysis and interpretation of data; 2) have been involved in drafting the manuscript or revising it critically for important intellectual content; and 3) have given final approval of the version to be published. Each author has participated sufficiently in the work to take public responsibility for appropriate portions of the content.

## Authors' information

Tang's group has been publishing image-based modeling work in recent years. For more information, please visit Tang's website: http://users.wpi.edu/~dtang/.

Dr. Yuan's group and their lab (Vascular Imaging Laboratory, University of Washington) have been developing MR imaging methods and have published extensively in this area. Website: http://www.rad.washington.edu/research/our-research/groups/vil.
